# A Novel Gudermannian Function-Driven Controller Architecture Optimized by Starfish Optimizer for Superior Transient Performance of Automatic Voltage Regulation

**DOI:** 10.3390/biomimetics11010007

**Published:** 2025-12-23

**Authors:** Davut Izci, Serdar Ekinci, Mostafa Jabari, Behçet Kocaman, Burcu Bektaş Güneş, Enver Adas, Mohd Ashraf Ahmad

**Affiliations:** 1 Department of Electrical and Electronic Engineering, Bursa Uludag University, 16059 Bursa, Turkey; davutizci@uludag.edu.tr (D.I.); enveradas@uludag.edu.tr (E.A.); 2 Applied Science Research Center, Applied Science Private University, Amman 11931, Jordan; 3 Department of Computer Engineering, Bitlis Eren University, 13100 Bitlis, Turkey; sekinci@beu.edu.tr; 4 Faculty of Electrical Engineering, Sahand University of Technology, Tabriz 51335, Iran; m_jabari97@sut.ac.ir; 5 Department of Electrical and Electronic Engineering, Bitlis Eren University, 13100 Bitlis, Turkey; bkocaman@beu.edu.tr; 6 Department of Computer Engineering, Istanbul Gedik University, 34876 İstanbul, Turkey; burcu.gunes@gedik.edu.tr; 7 Faculty of Electrical and Electronics Engineering Technology, University Malaysia Pahang Al-Sultan Abdullah, Pekan 26600, Pahang, Malaysia

**Keywords:** automatic voltage regulator, Gudermannian function-based PID controller, electrical power system, time response, starfish optimization algorithm

## Abstract

This paper proposes a Gudermannian function-based proportional–integral–derivative (G-PID) controller to enhance the transient performance of automatic voltage regulator (AVR) systems operating under highly dynamic conditions. By embedding the smooth and bounded nonlinear mapping of the Gudermannian function into the classical PID structure, the proposed controller improves adaptability to large signal variations while effectively suppressing overshoot. The controller parameters are optimally tuned using the starfish optimization algorithm (SFOA), which provides a robust balance between exploration and exploitation in nonlinear search spaces. Simulation results demonstrate that the SFOA-optimized G-PID controller achieves superior transient performance, with a rise time of 0.0551 s, zero overshoot, and a settling time of 0.0830 s. Comparative evaluations confirm that the proposed approach outperforms widely used optimization algorithms (particle swarm optimization, grey wolf optimizer, success history-based adaptive differential evolution with linear population size, and Kirchhoff’s law algorithm) and advanced AVR control schemes, including fractional-order and higher-order PID-based designs. These results indicate that the proposed SFOA optimized G-PID controller offers a computationally efficient and structurally simple solution for high-performance voltage regulation in modern power systems.

## 1. Introduction

The continuous and reliable delivery of electrical energy in modern power systems depends critically on precise voltage regulation and stable dynamic behavior. As electrical grids evolve toward increasingly heterogeneous, interconnected, and renewable-rich infrastructures, the task of maintaining voltage quality has become substantially more demanding. Automatic voltage regulators (AVRs) play a central role in meeting these requirements, as they ensure that the terminal voltage of synchronous generators remains within the desired bounds despite rapid changes in loading conditions, disturbances, and the inherent nonlinearities of large-scale electrical networks [1,2,3]. Maintaining a fast response, achieving minimal overshoot, and ensuring stable operation across a wide range of operating conditions have therefore become fundamental objectives in contemporary AVR research. These evolving demands have motivated the ongoing development of intelligent and adaptive control designs that can surpass the performance limitations of conventional approaches.

Historically, AVR systems have relied on proportional–integral–derivative (PID) controllers due to their structural simplicity, ease of implementation, and dependable performance around nominal or linearized operating points [1]. Yet, recent studies have repeatedly shown that traditional PID regulators (especially those tuned via classical procedures) struggle to meet the stringent performance expectations of modern power grids [4,5,6,7]. The primary issue arises from the fixed-gain nature of the controller, which is optimized under restrictive modeling assumptions. When subjected to nonlinear operation, significant parameter variation, and fast transient disturbances (conditions widely observed in today’s renewable-integrated grids) the fixed PID gains often fail to provide adequate robustness, speed, and damping [7,8,9]. This structural constraint has increasingly motivated the search for control laws capable of embedding adaptability and nonlinear compensation directly within their formulation, thus enabling a more resilient response under realistic system variability.

In this context, research since 2020 has explored a broad range of advanced control architectures, many of which increase structural flexibility by enhancing or extending the classical PID framework. Fractional-order PID (FOPID) controllers, for instance, introduce two additional degrees of freedom (λ and μ) that provide finer control over the system’s frequency and time responses and have demonstrated superior robustness against parametric uncertainties [10,11]. Other studies have pursued cascaded or hybrid designs, including combinations of fuzzy logic and fractional calculus (e.g., Fuzzy FOPI+FOPD+I) [12] or highly augmented cascaded structures incorporating multiple higher-order derivatives such as RPIDD^2^-FOPI [13]. These complex methods have effectively improved transient metrics, particularly settling time and overshoot reduction, but at the cost of considerable implementation difficulty and a dramatic increase in the dimensionality of the parameter-tuning problem [10,12]. The rising structural complexity of these architectures has also driven interest in alternative strategies that incorporate nonlinear analytical functions directly within the controller to achieve adaptive behavior with far less computational and design overhead [14,15]. By embedding a suitable nonlinear transformation of the error or the internal gains, such controllers can respond more effectively to rapid voltage deviations, thereby offering an attractive balance between robustness and structural simplicity [14].

The work presented in this paper is positioned precisely within this emerging class of functionally nonlinear control frameworks. A new controller the Gudermannian function [16]-based PID (G-PID) is proposed, marking the first integration of the Gudermannian function into a PID-type AVR structure. The Gudermannian function provides a mathematically elegant and smooth nonlinear mapping that links circular and hyperbolic functions without the use of complex numbers [17]. Owing to its bounded, sigmoid-like shape, it is particularly suitable for enhancing gain adaptability and mitigating aggressive control actions, ultimately contributing to improved transient behavior and enhanced robustness in systems exhibiting nonlinear dynamics [16]. Embedding this analytically derived nonlinearity within a conventional PID framework enables the controller to maintain the structural simplicity of PID while gaining an inherent ability to compensate for nonlinearities without relying on the extensive rule bases required in fuzzy logic systems or the computational burden associated with fractional calculus [16]. Because such a functional extension has not previously been utilized in AVR control, the G-PID structure represents a novel contribution, offering a distinct and theoretically grounded pathway toward improved AVR performance.

The introduction of nonlinear elements into the controller structure, however, increases the complexity of the associated tuning problem. Whether dealing with FOPID controllers, cascaded PID, or nonlinear PID formulations, the resulting search space is almost always multimodal, non-convex, and often high-dimensional [18,19,20]. Consequently, classical gradient-based optimization becomes ineffective in identifying globally optimal parameters. Over the last decade, population-based metaheuristic algorithms and other evolutionary schemes [21,22,23,24] have been widely applied in AVR tuning problems and fractional-order control optimization [25]. Although these algorithms have demonstrated reasonable success, they are still susceptible to premature convergence, particularly when the tuning landscape contains many local minima, as is typical for highly structured controllers with six or more parameters [26]. Premature convergence results in suboptimal controller gains that may perform well near specific operating conditions but fail to deliver the robustness and speed necessary across the full range of AVR dynamics. The increasing structural complexity of advanced controllers thus underscores the necessity for newer optimization strategies capable of maintaining a strong balance between exploration and exploitation while ensuring stable convergence toward the global optimum [27,28].

In response to this need, the present study employs the starfish optimization algorithm (SFOA) as the tuning mechanism for the proposed G-PID controller. SFOA is a recently introduced bio-inspired optimization method modeled on the regenerative, adaptive, and cooperative behaviors of starfish [5]. Although numerous metaheuristic algorithms have been successfully applied to AVR controller tuning, the selection of an appropriate optimizer remains problem dependent and closely linked to the structure of the control law being optimized. In the present study, the tuning problem involves a nonlinear controller architecture with multiple interdependent parameters, resulting in a multimodal and non-convex search landscape where premature convergence is a common challenge for many classical and recently proposed metaheuristics. The SFOA was selected in this work due to its distinctive search mechanisms, which combine dimension-adaptive exploration, cooperative exploitation, and a regeneration strategy designed to preserve population diversity. These characteristics enable SFOA to effectively navigate complex optimization landscapes and reduce the likelihood of stagnation in local optima. Recent benchmark studies have demonstrated that SFOA exhibits competitive or superior convergence stability compared with a large number of established metaheuristic algorithms, particularly in high-dimensional and nonlinear optimization problems. Given the nonlinear nature of the Gudermannian-based PID controller and the strong coupling between its parameters, SFOA provides a suitable optimization framework capable of ensuring reliable global tuning while maintaining computational efficiency. Therefore, the adoption of SFOA in this study is motivated by its structural compatibility with the optimization challenges posed by advanced AVR controller design rather than by arbitrary algorithm selection.

The performance expectations for modern AVR systems have become exceptionally demanding. Recent studies frequently pursue the simultaneous achievement of minimal rise time, ultra-fast settling time, and zero or near-zero overshoot (OS%) which is a combination that defines the upper tier of AVR performance benchmarks [29]. However, achieving all three objectives concurrently remains a persistent challenge in the literature. While many advanced control structures have succeeded in minimizing settling time or eliminating overshoot, they often do so using highly complex architectures such as cascaded RPIDD^2^-FOPI, PI^λ1^I^λ2^D^μ1^D^μ2^, or FOPIDD^2^ controllers, each of which involve substantial implementation and tuning burdens [30,31,32,33,34]. Other methods introducing fuzzy or hybrid control achieve robustness but at the expense of slower settling times or non-zero overshoot [30,31,32,33,34]. Table 1 in this study consolidates the performance characteristics, advantages, and shortcomings of ten prominent metaheuristic-optimized AVR controllers. This comparison highlights a clear research gap: although state-of-the-art controllers achieve impressive transient behavior, they often require structural intricacy that limits their practicality.

The proposed SFOA-optimized G-PID controller directly addresses this gap. Its aim is to demonstrate that a structurally simple yet functionally enhanced PID controller (augmented by a smooth analytic nonlinearity and tuned by a high-performance new-generation metaheuristic) can match or exceed the transient performance of significantly more complex control architectures. In particular, the target is to achieve near-zero overshoot and ultra-fast settling times similar to those produced by cascaded or fractional-order methods, but without resorting to multi-layered structures or fractional differentiation. By embedding the Gudermannian function into the control loop and optimizing its scaling parameter alongside the PID gains, the controller acquires the adaptability necessary to manage nonlinearities while retaining a compact and easily implementable form. This combination of analytical innovation and advanced optimization is presented here for the first time in AVR control.

To clearly highlight the originality and technical contributions of this study, the main contributions are summarized as follows:

- A novel G-PID controller is proposed for AVR systems, representing the first integration of the Gudermannian function into a PID-type voltage regulation framework.
- A smooth and bounded nonlinear mapping is analytically embedded within the classical PID structure, enhancing adaptability to large voltage deviations while suppressing overshoot and preserving structural simplicity.
- The SFOA is employed for optimal controller tuning, marking its first application to AVR control problems and enabling effective global optimization in a nonlinear and multimodal search space.
- Extensive simulation and statistical analyses are conducted, demonstrating that the proposed SFOA-optimized G-PID controller achieves superior transient performance in terms of rise time, settling time, and overshoot compared with widely used metaheuristic-optimized controllers.
- A comprehensive benchmarking study against recent state-of-the-art AVR controllers (2024–2025) confirms that the proposed approach delivers competitive or superior performance while avoiding the structural and computational complexity of fractional-order, fuzzy, and cascaded control designs.

## 2. Overview of Starfish Optimization Algorithm

The starfish optimization algorithm (SFOA) is a recently introduced bio-inspired metaheuristic that models the natural behaviors of starfish, including exploration, preying, and regeneration. SFOA integrates a hybrid exploration mechanism with an efficient exploitation strategy, enabling strong performance in nonlinear and multimodal optimization problems. These characteristics make SFOA well-suited for controller tuning tasks, including the optimization of nonlinear Gudermannian-based PID controllers in AVR systems. Starfish exhibit several distinctive behaviors that translate naturally into optimization concepts:

- Exploratory multi-arm sensing: A typical starfish has five flexible arms, each capable of moving independently to sense environmental conditions. This behavior inspires a multi-directional search pattern during the exploration phase.
- Coordinated preying behavior: When hunting, starfish simultaneously adjust arm positions and rely on mutual feedback. This cooperative movement motivates SFOA’s two-directional exploitation strategy.
- Regeneration capability: Starfish can detach and regenerate an arm when threatened. This mechanism inspires a diversity-enhancing regeneration step used to prevent premature convergence.

These biological principles are embedded in the mathematical structure of SFOA. SFOA operates on a population of candidate solutions:

(1) $$X=\left\{X_{1},X_{2},\ldots ,X_{N}\right\}\in R^{N\times D}$$

where N is the population size and D is the dimensionality of the optimization problem. Each iteration of SFOA consists of exploration and exploitation, selected with equal probability $\left(G_{P}=0.5\right)$. A key feature of SFOA is its dimension-adaptive hybrid exploration strategy. A five-dimensional search pattern updates five randomly selected dimensions, mimicking the five arms of starfish. For high-dimensional problems (*D* > 5), the following definition is used:

(2) $$Y_{i,p}^{\left(t\right)}=X_{i,p}^{\left(t\right)}\pm \alpha_{1}\left(X_{best,p}^{\left(t\right)}-X_{i,p}^{\left(t\right)}\right)\Phi \left(\theta \right)$$

where $\alpha_{1}=\left(2r-1\right)\pi$, $\theta =\frac{\pi }{2}\left(1-\frac{t}{T_{max}}\right)$, Φ(θ)∈sin(θ), cos(θ). This structure enhances exploration capability and reduces computational load. For low-dimensional problems (*D* ≤ 5), a unidimensional search pattern is used:

(3) $$Y_{i,p}^{\left(t\right)}=E_{t}X_{i,p}^{\left(t\right)}+A_{1}\left(X_{k_{1},p}^{\left(t\right)}-X_{i,p}^{\left(t\right)}\right)+A_{2}\left(X_{k_{2},p}^{\left(t\right)}-X_{i,p}^{\left(t\right)}\right)$$

with $A_{1},A_{2}\in \left(-1,1\right)$ and the adaptive energy function.

(4) $$E_{t}=\left(\frac{T_{max}-t}{T_{max}}\right)cos\left(\theta \right)$$

This facilitates controlled exploration motion when the search space is small. The exploitation phase consists of two components (preying behavior and regeneration behavior), In preying behavior, SFOA applies a parallel two-directional search:

(5) $$Y_{i}^{\left(t\right)}=X_{i}^{\left(t\right)}+r_{1}d_{m_{1}}+r_{2}d_{m_{2}}$$

where $d_{m}=X_{best}-X_{m}$. This mechanism allows forward and backward movement simultaneously, improving escape from local optima. In regeneration behavior, only the worst-performing starfish (solution) undergoes regeneration:

(6) $$Y_{i}^{\left(t\right)}=e^{-\frac{t\times N}{T_{max}}}X_{i}^{\left(t\right)}$$

This slow, controlled reinitialization restores diversity and prevents stagnation. Table 2 shows pseudo code for SFOA optimization method.

## 3. Modelling and Description of AVR System

### General Overview of AVR System

The AVR model consists of four main components namely amplifier, exciter, generator and sensor. Each of these subsystems is represented by an individual transfer function, while the effects of saturation and nonlinearity are neglected, as modeled in MATLAB/Simulink (2025b). The corresponding transfer function equations, along with their gains, time constants, and operational limits, are summarized in Table 3.

**Figure 1:**
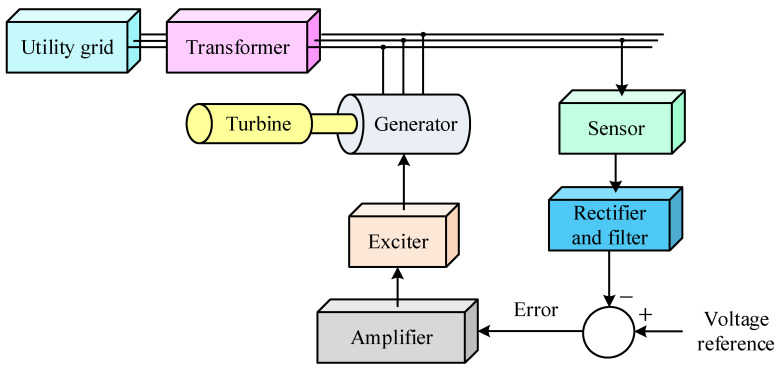
Physical arrangement of the AVR within the overall power generation setup.

A broader representation of the system is shown in Figure 1, which highlights the physical arrangement of the AVR within the overall power generation setup. The turbine drives the generator, whose output is delivered to the utility grid through a transformer. The sensor continuously monitors the generator output voltage and feeds it back through a rectifier and filter to be compared with the voltage reference. The AVR system, through this feedback loop, ensures stable voltage regulation and reliable operation of the synchronous generator under varying load and operating conditions [38].

The transfer function of an uncontrolled AVR system is given in Equation (7):

(7) $$G\left(s\right)=\frac{0.1s+10}{0.0004s^{4}+0.0454s^{3}+0.555s^{2}+1.51s+11}$$

**Figure 2:**
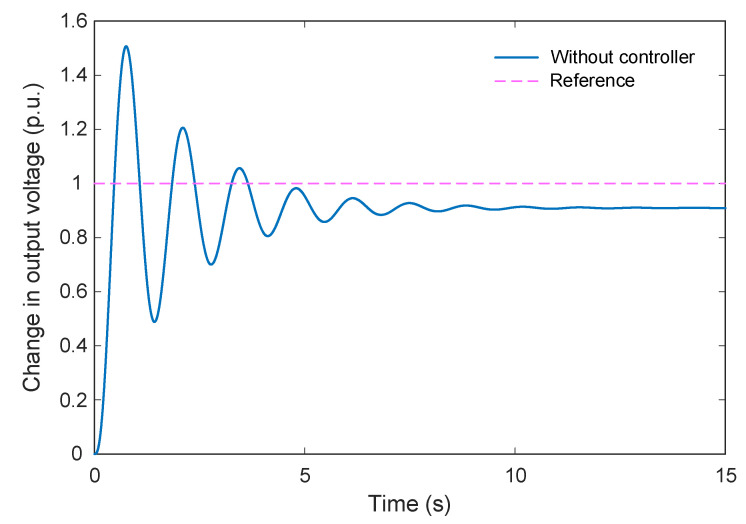
Step response on AVR system for without controller mode.

The step response of the AVR system is presented in Figure 2. As observed from the figure, the maximum overshoot reaches approximately 1.51 p.u., with a rise time of 0.261 s and a settling time of 6.99 s. These results indicate that the system exhibits considerable overshoot and noticeable oscillatory behavior, reflecting the need for further tuning to enhance its transient stability and damping performance.

## 4. Proposed Novel Control Method

### 4.1. Conceptual Basis of the Gudermannian Function

**Figure 3:**
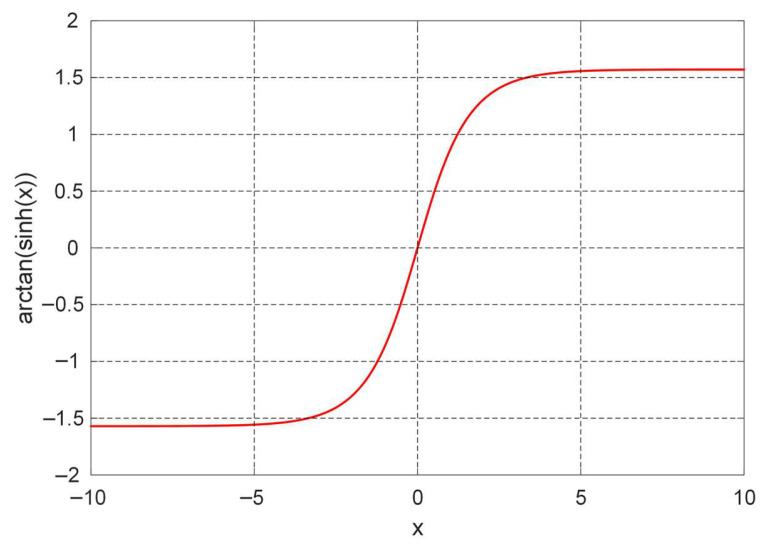
Numerical plot of the Gudermannian function.

The Gudermannian function, denoted as gd(x) is a special transcendental function of notable importance in mathematical analysis. It establishes a purely real-valued correspondence between the hyperbolic and circular functions without involving complex numbers. In essence, it provides a continuous and real mapping that connects the geometry of the hyperbola with that of the circle. The function acts as a transformation from a hyperbolic argument x to a circular angle gd(x), whose value lies within $\left[-\frac{\pi }{2},\frac{\pi }{2}\right]$. The fundamental identity expressing this connection is given by definition in Equation (8). Figure 3 illustrates this function for −10≤x≤10.

(8) gd(x)=arctan(sinh(x))

### 4.2. A Novel Control Approach for AVR System: Gudermannian Function-Based PID Controller

**Figure 4:**
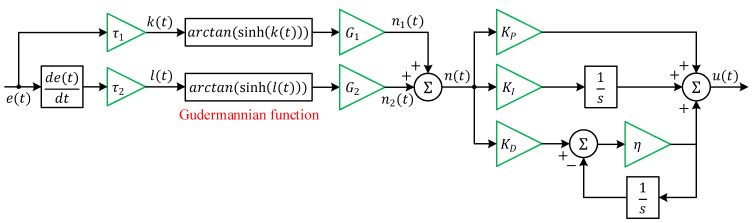
System architecture of the Gudermannian function-based PID controller.

The architecture illustrated in Figure 4 defines the mathematical framework of the Gudermannian function-based PID (G-PID) controller. In this structure, the control output *u*(*t*), is generated from a nonlinear composite signal *n*(*t*), through proportional, integral, and filtered derivative branches. The nonlinear stage employs the Gudermannian transformation, which establishes a smooth, bounded relationship between the error signal and its response, improving transient behavior and robustness under system nonlinearity. Let *e*(*t*) represent the instantaneous voltage error and $\;\dot{e}\left(t\right)=\frac{de\left(t\right)}{dt}$ its time derivative. Two scaled inputs are formed:

(9) $$k\left(t\right)=\tau_{1}e\left(t\right)$$

(10) $$l\left(t\right)=\tau_{2}\dot{e}\left(t\right)$$

where $\tau_{1}$ and $\tau_{2}$ are positive scaling coefficients that render the arguments dimensionless. The nonlinear mapping is implemented using the Gudermannian function gd(x)=arctan(sinh(x)). which is smooth, monotonic, and bounded in the interval, $\left(-\frac{\pi }{2},+\frac{\pi }{2}\;\right)$. Applying this transformation yields the nonlinear outputs:

(11) $$n_{1}\left(t\right)=G_{1}arctan\left(\sinh\left(\tau_{1}e\left(t\right)\right)\right)$$

(12) $$n_{1}\left(t\right)=G_{2}arctan\left(\sinh\left(\tau_{2}\dot{e}\left(t\right)\right)\right)$$

and the combined nonlinear signal driving the PID core:

(13) $$n\left(t\right)=n_{1}\left(t\right)+n_{2}\left(t\right)=G_{1}arctan\left(\sinh\left(\tau_{1}e\left(t\right)\right)\right)+G_{2}arctan\left(\sinh\left(\tau_{2}\dot{e}\left(t\right)\right)\right)$$

The G-PID controller output is the superposition of the proportional, integral, and filtered derivative actions which are given as follows.

(14) $$PID\left(s\right)=\frac{U(s)}{N(s)}=K_{P}+\frac{K_{I}}{s}+K_{D}\frac{\eta s}{\eta +s}$$

### 4.3. Constraint of Optimization Problem and Definition of Cost Function

To determine the optimal parameter set of the proposed Gudermannian function-based PID controller, an unconstrained optimization problem is formulated with bounded decision variables and a scalar performance index. The objective of the optimizer is to minimize the time-domain error characteristics of the AVR system while ensuring a balanced contribution of transient and steady-state metrics.

The cost function adopted in this study follows the weighted performance formulation proposed in [39,40]. It simultaneously accounts for overshoot, steady-state accuracy, and transient response characteristics. The cost function is defined as:

(15) $$CF=\left(1-e^{-\xi }\right)\left(\frac{m_{os}+e_{ss}}{100}\right)+e^{-\xi }\left(t_{s}-t_{r}\right)$$

where $m_{os}$, $e_{ss}$, $t_{s}$, $t_{r}$ and ξ are the percent overshoot (%), steady-state error (%) evaluated at t=100 s, the settling time based on the ±2% tolerance band, the rise time, the balance factor, set to ξ=1 as recommended in [41]. The exponential weighting term $e^{-\xi }$ provides a continuous trade-off between overshoot/steady-state accuracy and speed of response. For ξ=1, the contribution of both performance groups becomes balanced, preventing the optimizer from converging to solutions with extremely fast but oscillatory responses, or overly slow but highly damped behaviors.

The optimization process searches for eight controller parameters that define the behavior of the G-PID structure, including the classical PID gains and the nonlinear mapping coefficients. The lower and upper bounds of the decision variables are listed in Table 4. These bounds ensure both the stability of the closed-loop system and the feasibility of the nonlinear transformation introduced by the Gudermannian function.

### 4.4. Utilization of the SFOA in the AVR Optimization Task

**Figure 5:**
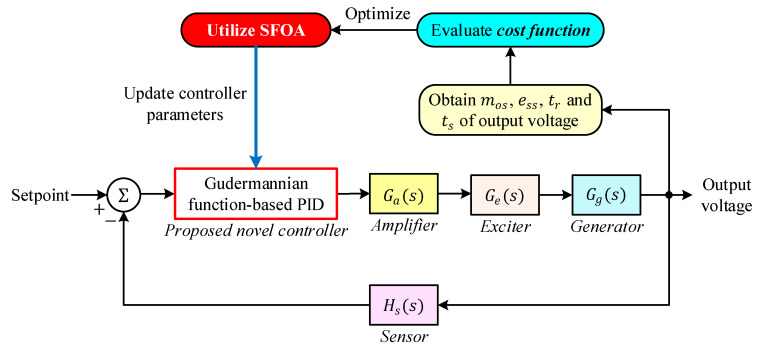
General schematic for proposed controller tuned by SFOA optimization method.

The SFOA is employed to determine the optimal set of parameters for the proposed Gudermannian function-based PID controller. The optimization framework operates in an outer-loop structure, where SFOA iteratively updates the controller parameters based on the performance of the closed-loop system. The complete optimization procedure is illustrated in Figure 5.

At each iteration of SFOA, a candidate solution represents a distinct parameter vector containing the PID gains $\left(K_{p},K_{I},K_{D}\right)$, as well as the nonlinear shaping coefficients of the Gudermannian transformation. These candidate parameters are applied to the controller block within the AVR model, and the closed-loop response to a unit step change in the reference voltage is simulated. From the resulting output voltage profile, four performance indices percent overshoot $\left(m_{os}\right)$, steady-state error $\left(e_{ss}\right)$, rise time $\left(t_{r}\right)$, and settling time $\left(t_{s}\right)$ are extracted. These metrics are then substituted into the cost function defined in Section 4.3, providing a scalar fitness value for the current candidate solution. SFOA subsequently adjusts the controller parameters by performing its biologically inspired search operations, thereby generating improved candidates with progressively lower cost function values. This iterative evaluation–update cycle continues until the termination criteria of SFOA are satisfied, resulting in a parameter set that minimizes the time-domain error while enhancing voltage regulation performance. Through this mechanism, SFOA autonomously tunes the proposed G-PID controller to achieve a fast, well-damped, and overshoot-free AVR response.

All simulations and optimization procedures presented in this study were conducted using the MATLAB/Simulink environment (version 2025b) on a Windows-based personal computer. The computational platform was equipped with a 12th Generation Intel^®^ Core™ i7-1260P processor operating at 2.10 GHz and 16.0 GB of RAM. This setup provided sufficient computational resources to ensure stable execution of the metaheuristic optimization algorithms and accurate time-domain simulations of the AVR system.

## 5. Simulation Results

### 5.1. Selected Optimization Algorithms

To evaluate the effectiveness of the proposed Gudermannian function-based PID controller, its optimized performance is compared with several well-established metaheuristic algorithms reported in recent literature. Five population-based optimizers are selected: the starfish optimization algorithm (SFOA) [42], Kirchhoff’s law algorithm (KLA) [43], success history-based adaptive differential evolution with linear population size (L-SHADE) [44], grey wolf optimizer (GWO) [7], and particle swarm optimization (PSO) [45]. These algorithms represent diverse classes of bio-inspired, physics-inspired, and evolution-based search mechanisms, ensuring a fair and comprehensive benchmarking environment.

For all optimizers, a common experimental setting is adopted to maintain consistency across the simulations. Each algorithm is executed for 25 independent runs, with a population size of 20 and a maximum of 100 iterations per run. These conditions ensure a balanced trade-off between computational cost and solution quality, while also providing statistically reliable performance indicators. Table 5 summarizes the control parameters used for each optimization algorithm. SFOA operates with its primary governing parameter $G_{P}$, whereas KLA does not require algorithm-specific coefficients. L-SHADE makes use of adaptive differential evolution parameters, specifically the crossover rate and scaling factor. In GWO, the convergence constant (a) decreases linearly from 2 to 0, guiding the transition between exploration and exploitation. PSO utilizes classical inertia weights and acceleration coefficients as defined in [45].

### 5.2. Statistical Analysis

**Figure 6:**
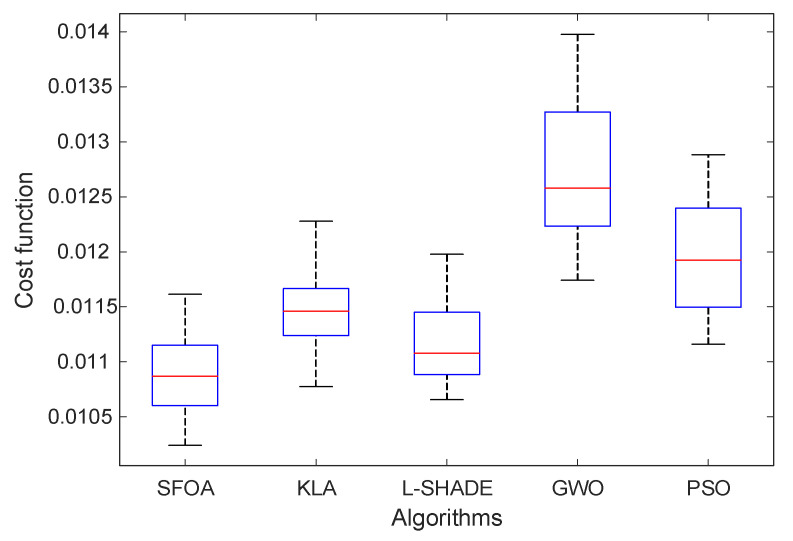
Boxplot distribution of the final cost function values obtained by SFOA, KLA, L-SHADE, GWO, and PSO.

A comprehensive statistical evaluation was performed to assess the reliability and robustness of the optimization algorithms over 25 independent runs. Figure 6 presents the boxplot distribution of the final cost function values obtained by SFOA, KLA, L-SHADE, GWO, and PSO. The spread, median, and extremal values depicted in the figure provide a clear comparative insight into the stability of each optimizer. As illustrated, SFOA exhibits the narrowest interquartile range and the lowest median cost value, indicating superior consistency and convergence reliability. In contrast, GWO produces the widest distribution, reflecting greater variability and a tendency to converge to suboptimal regions. PSO also demonstrates moderate variability, whereas KLA and L-SHADE achieve improved stability but do not match the performance of SFOA in terms of minimum and average cost.

The quantitative results are reported in Table 6. SFOA achieves the smallest, best value $\left(1.0241\times 10^{-2}\right)$, the lowest average value $\left(1.0888\times 10^{-2}\right)$, and the smallest standard deviation $\left(4.0055\times 10^{-4}\right)$ among all methods. These indicators confirm that SFOA not only finds higher-quality solutions but also maintains strong robustness across repeated trials. L-SHADE and KLA follow with comparable average performance, though with slightly higher variability. GWO yields the weakest results with the highest average and worst-case cost values, consistent with the larger spread observed in the boxplot. The combination of visual and numerical analysis clearly demonstrates that SFOA provides the most reliable and stable optimization behavior for tuning the proposed G-PID controller.

### 5.3. Change in Cost Function

**Figure 7:**
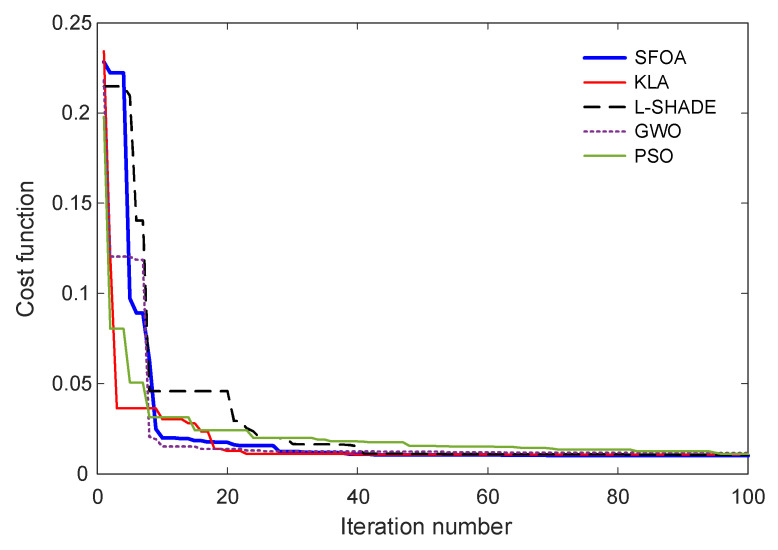
Convergence curves of SFOA compared with benchmark algorithms.

The convergence behavior of the optimization algorithms is illustrated in Figure 7, which presents the evolution of the cost function values over 100 iterations. This analysis reveals the relative efficiency, search dynamics, and stability of the candidate optimizers when tuning the Gudermannian function-based PID controller. As observed in Figure 7, all algorithms demonstrate a rapid decrease in the cost function during the early stages of the search process, indicating effective exploration of the solution space. Among them, SFOA achieves one of the fastest drops within the first 10 iterations, quickly converging toward a near-optimal region. KLA also shows an abrupt decline at the outset but settles at a higher cost level compared with SFOA. L-SHADE exhibits a delayed yet steady descent, stabilizing at a similar convergence level to KLA. In contrast, GWO presents slower convergence and remains at higher cost values throughout most of the optimization process, reflecting less efficient balance between exploration and exploitation. PSO initially decreases sharply but demonstrates more oscillatory behavior before reaching its final convergence level.

The optimized parameters obtained by each algorithm are presented in Table 7. The results show that SFOA yields a distinctive parameter configuration, with a notably large value of η and comparatively moderate nonlinear shaping coefficients $\left(\tau_{1},G_{1},\tau_{2},G_{2}\right)$. This parameter combination effectively enhances the nonlinear transformation characteristics of the G-PID controller, contributing to its superior performance observed in earlier sections. KLA and L-SHADE converge to parameter sets with similar magnitudes, consistent with their comparable cost values. GWO produces the most divergent parameter values, which aligns with its noticeably weaker convergence pattern. PSO identifies relatively moderate gain values but does not match the overall performance of SFOA. The convergence curves and final parameter sets demonstrate that SFOA provides the most efficient and stable optimization behavior. Its ability to rapidly reduce the cost function and locate high-quality solutions validates its suitability for tuning the proposed nonlinear PID controller in AVR applications.

### 5.4. Step Response

**Figure 8:**
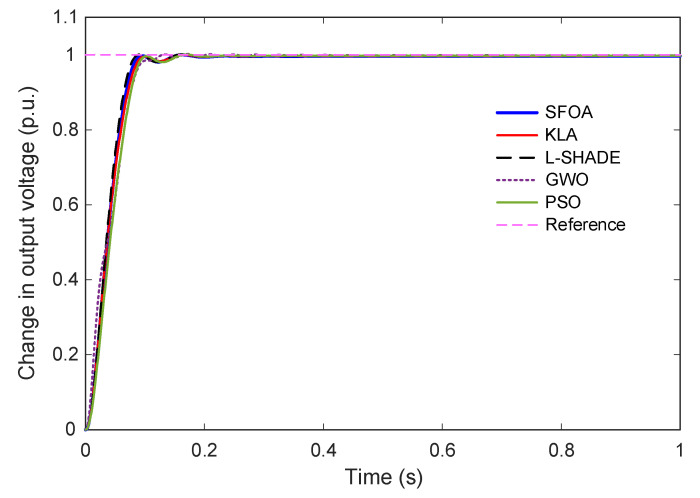
Time-domain response of the AVR system tuned by SFOA and benchmark algorithms.

**Figure 9:**
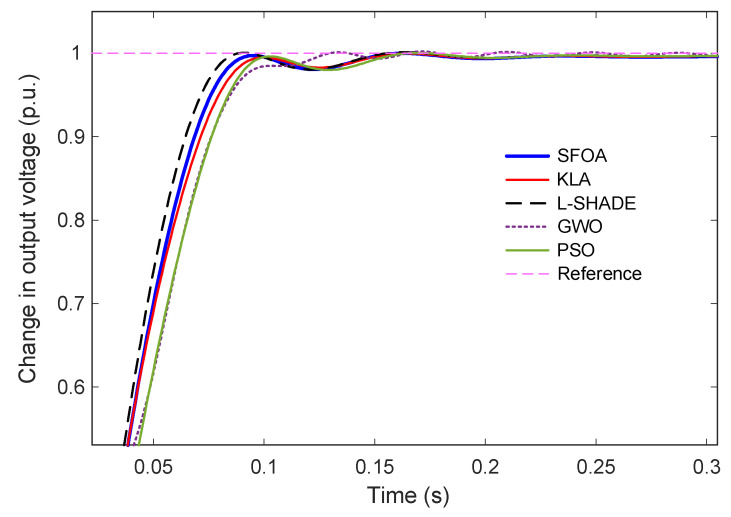
Zoomed-in step response showing early transient behavior for SFOA, KLA, L-SHADE, GWO, and PSO.

The dynamic performance of the AVR system under the proposed Gudermannian function-based PID controller, optimized by the selected metaheuristic algorithms, is evaluated through the unit step response shown in Figure 8. A magnified view of the transient interval (0–0.3 s) is provided in Figure 9 to highlight early-time differences among the controllers. Figure 8 illustrates that all optimized controllers achieve satisfactory tracking performance, converging smoothly to the reference value of 1 p.u. without steady-state deviation. However, the response profiles exhibit notable distinctions in the speed of convergence and degree of overshoot. The trajectories associated with SFOA and KLA demonstrate fast response with minimal oscillations, while L-SHADE, GWO, and PSO show larger overshoot and slower stabilization.

The zoomed-in view in Figure 9 further clarifies these differences. The SFOA-tuned controller exhibits a rapid rise with no observable overshoot, indicating a highly damped and well-regulated transient characteristic. KLA produces a similar zero-overshoot response but with a slightly longer rise time and settling time. L-SHADE and PSO reach the reference more quickly, but both exhibit non-negligible overshoot, particularly in the early transient phase. GWO yields the slowest rise time and the largest overshoot among all algorithms, consistent with its inferior convergence behavior discussed in earlier subsections.

Quantitative metrics extracted from the responses are summarized in Table 8. SFOA achieves the best overall performance, with the smallest settling time (0.0830 s), zero overshoot, and a competitive rise time of 0.0551 s. L-SHADE attains the shortest rise time (0.0511 s) but at the expense of overshoot. KLA provides a balanced and stable response with slightly slower dynamics than SFOA. GWO and PSO, while stable, fall short in terms of both overshoot suppression and settling speed. Finally, these results confirm that the SFOA-optimized G-PID controller delivers the most desirable transient characteristics, achieving a fast, smooth, and entirely overshoot-free voltage regulation response.

### 5.5. Comparison of Well-Known Control Design Methods Reported in the Literature

To further validate the superiority of the proposed Gudermannian function-based PID controller, its performance is compared with several advanced and widely cited AVR control strategies. These benchmark controllers include TSA-optimized PID [46], AOA-optimized PID-F [47], HGSO-optimized FOPID [48], WOA-optimized PIDA [49], and FWWOA-optimized PIDD^2^ [50]. Their corresponding transfer functions and optimal parameter values are summarized in Table 9.

**Figure 10:**
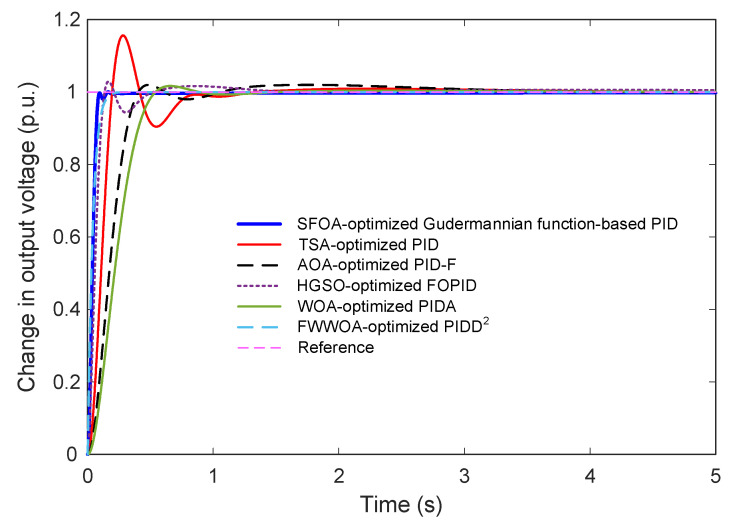
Transient behavior of the AVR system for the proposed and existing optimized controllers.

**Figure 11:**
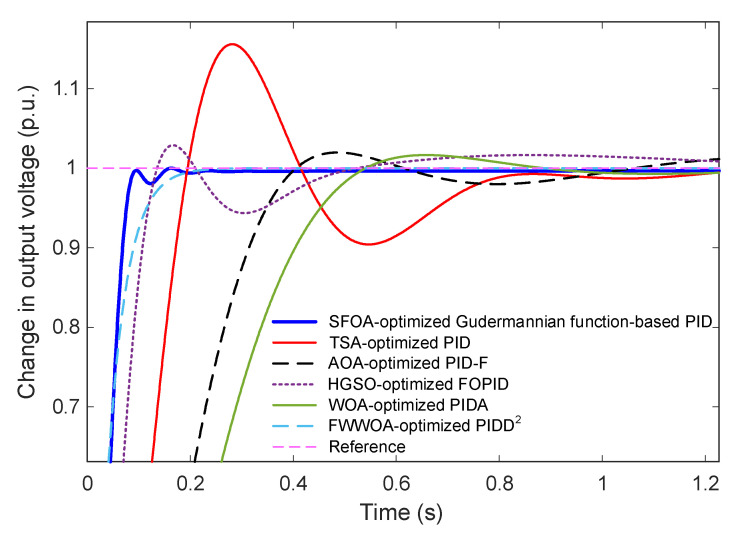
Zoomed-in comparison of early transient behavior for SFOA-optimized Gudermannian PID and existing optimized controllers.

Figure 10 and Figure 11 present the comparative step responses over a 5-s interval and a zoomed-in transient region (0–1.2 s), respectively. The results reveal substantial performance differences among the controllers. The SFOA-optimized Gudermannian PID demonstrates the fastest rise with smooth convergence and no observable overshoot, confirming its strong damping capability and well-regulated transient behavior. In contrast, the TSA-optimized PID exhibits a significantly oscillatory response with a large overshoot exceeding 15%, resulting in slow stabilization. The AOA-optimized PID-F and HGSO-optimized FOPID achieve improved damping, but still present noticeable overshoot and slower convergence compared to the proposed controller. The WOA-optimized PIDA shows marked sluggishness, with a slow rise and long settling time, whereas the FWWOA-optimized PIDD^2^ performs better but still cannot eliminate overshoot entirely.

The quantitative performance indices listed in Table 10 reinforce these observations. The proposed controller achieves the smallest settling time (0.0830 s), zero overshoot, and a competitive rise time of (0.0551 s), outperforming all benchmark methods across all metrics. Among existing approaches, the FWWOA-optimized PIDD^2^ achieves moderate performance with relatively low overshoot but remains noticeably inferior in rise and settling time. The classical TSA-PID and PIDA-based controller perform the worst due to excessive oscillations and delayed convergence. Finally, these comparative results show that the proposed Gudermannian function-based PID controller delivers a substantially improved transient and steady-state response compared to state-of-the-art optimization-based AVR controllers. Its ability to ensure overshoot-free operation while maintaining exceptional speed highlights its effectiveness for high-performance voltage regulation applications.

### 5.6. Comparison with State-of-the-Art AVR Studies

To position the proposed Gudermannian function-based PID controller within the broader landscape of recent AVR research, a comparative analysis is conducted using ten state-of-the-art control strategies published between 2024 and 2025. These methods, summarized in Table 11, represent a diverse range of control paradigms, including fuzzy-PID hybrids, fractional-order PID controllers, sliding-mode control, high-order differential feedback, and bio-inspired optimization-based PID variants. The comparison aims to evaluate the proposed controller’s relative performance in terms of rise time, overshoot, and settling time.

**Figure 12:**
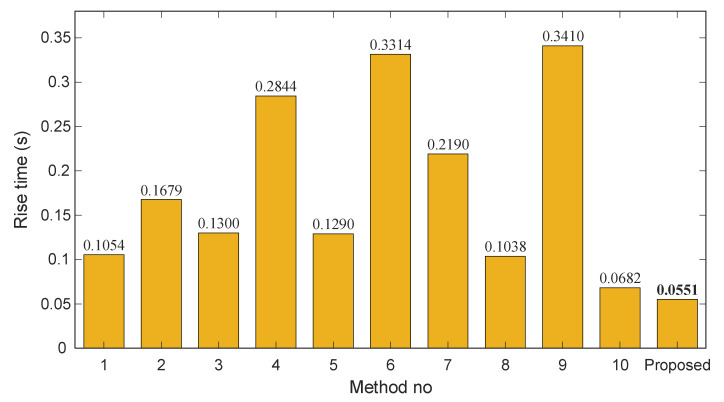
Bar chart of rise time (s) across existing methods and the proposed controller.

**Figure 13:**
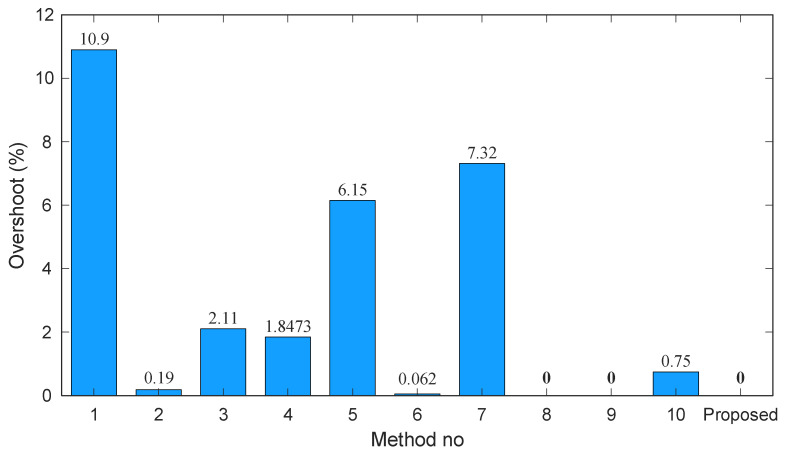
Bar chart of overshoot (%) across existing methods and the proposed controller.

**Figure 14:**
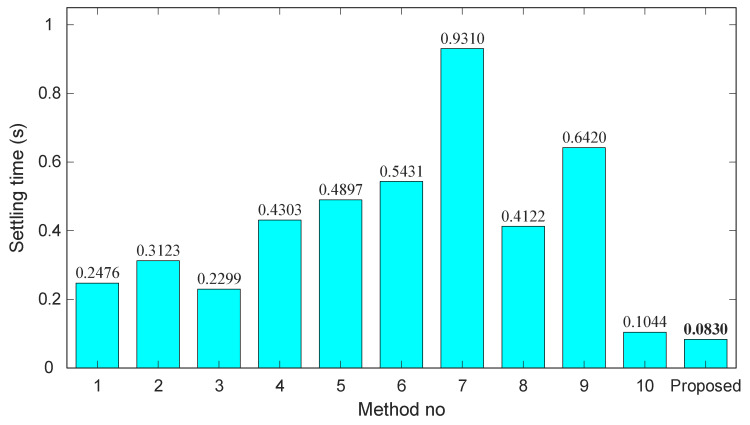
Bar chart of settling time (s) across existing methods and the proposed controller.

Figure 12, Figure 13 and Figure 14 depict the rise time, overshoot, and settling time for each method, with the proposed approach included for reference. As shown in Figure 12, the rise times of the existing methods vary significantly, ranging from approximately 0.07 s to over 0.34 s. In contrast, the proposed controller achieves the smallest value (0.0551 s), demonstrating a considerably faster dynamic response than all competing techniques. This rapid rise underscores the strong tracking capability introduced by the Gudermannian-based nonlinear transformation.

The overshoot characteristics in Figure 13 further highlight the effectiveness of the proposed method. Several controllers, particularly those incorporating fractional-order dynamics or advanced optimization heuristics exhibit moderate to severe overshoot levels, with values exceeding 6% in methods 5 and 7 and reaching as high as 10.9% in method 1. Other approaches achieve improved damping but still fail to eliminate overshoot. The proposed controller, however, maintains zero overshoot, indicating exceptional transient regulation and robustness to rapidly changing voltage conditions.

Settling time results, presented in Figure 14, reinforce this advantage. Existing approaches report settling times between 0.10 s and 0.93 s, with method 7 showing the slowest convergence. The proposed controller attains the minimum settling time of 0.0830 s, outperforming even the fastest state-of-the-art designs. This result confirms that the combination of the Gudermannian nonlinearity and SFOA tuning yields a highly stable and quickly converging closed-loop response.

Overall, across all three key performance metrics such as, rise time, overshoot, and settling time, the proposed SFOA-optimized Gudermannian PID controller consistently surpasses the capabilities of the most recent and advanced AVR control methodologies. This comparison provides strong evidence of its superiority and suitability for high-performance voltage regulation applications in modern power systems.

### 5.7. Discussion

The simulation results presented in Section 5.1, Section 5.2, Section 5.3, Section 5.4, Section 5.5 and Section 5.6 collectively demonstrate the superiority of the proposed SFOA-optimized Gudermannian function-based PID controller over a wide spectrum of benchmark methods. The analysis covers algorithmic behavior, transient performance, and comparative evaluations with both classical and cutting-edge AVR controllers. Several important insights emerge from these findings.

First, the statistical evaluation of the optimization algorithms highlights the effectiveness of SFOA in navigating the multidimensional parameter space of the proposed nonlinear controller. As shown in the boxplots and numerical statistics, SFOA achieves the lowest mean and best cost function values with the smallest standard deviation, indicating strong convergence stability and reduced sensitivity to initial conditions. This advantage is further reinforced by the cost function evolution curves, where SFOA consistently converges more rapidly than KLA, L-SHADE, PSO, and GWO. These results suggest that the exploration–exploitation balance inherent to SFOA aligns well with the nonlinear and highly coupled structure of the G-PID controller.

Second, the transient response characteristics of the optimized controllers reveal clear performance distinctions. The proposed method achieves zero overshoot and extremely fast settling dynamics, setting it apart from the other optimized controllers. Unlike L-SHADE and PSO, which deliver competitive rise times but exhibit oscillatory or overshooting behavior, the proposed controller maintains exceptional damping without sacrificing speed. Similarly, KLA and GWO deliver slower or more oscillatory responses, reflecting the limitations of their search strategies when tuning nonlinear control structures. The ability of the proposed method to combine rapid tracking with smooth transient behavior highlights the effectiveness of the Gudermannian nonlinear mapping, which acts as a bounded, smooth gain adaptation mechanism during large signal deviations.

Third, comparisons with well-established AVR designs from the literature underline the generalizability and practical significance of the proposed approach. Against a broad set of optimization-based PID, FOPID, PID-F, PIDA, and higher-order controllers, the proposed method consistently achieves the best rise time, zero overshoot, and the shortest settling time. This is particularly noteworthy given the complexity and computational cost associated with some fractional-order and high-order differential controllers. The proposed design, despite maintaining a PID-type structure, provides superior dynamic performance, demonstrating that the Gudermannian transformation enriches the controller behavior without requiring structural complexity.

Finally, the comparison with state-of-the-art AVR designs from 2024–2025 establishes the robustness of the proposed method across a wider methodological spectrum. Controllers based on fuzzy logic, sliding-mode control, fractional-order dynamics, and bio-inspired optimizers show diverse levels of performance, with several suffering from large overshoot, sluggish settling, or inconsistent dynamics. In contrast, the proposed controller achieves the most balanced and consistently high-performing results across all metrics. Its zero-overshoot characteristic is particularly valuable for AVR applications, where voltage excursions can propagate through the network and impact stability and protection mechanisms.

Overall, the discussion highlights that the combination of the Gudermannian nonlinear transformation with SFOA-based parameter tuning provides a powerful, computationally efficient, and highly robust control strategy. The proposed controller not only surpasses traditional and fractional-order PID designs but also outperforms advanced state-of-the-art AVR control methods, positioning it as a strong candidate for deployment in modern power systems requiring fast, stable, and reliable voltage regulation.

## 6. Conclusions and Future Directions

This study introduced a G-PID controller for AVR systems and demonstrated its effectiveness when optimally tuned using the SFOA. By embedding a smooth and bounded nonlinear transformation into the conventional PID framework, the proposed controller was shown to enhance transient stability, suppress overshoot, and improve damping characteristics under large signal variations. These improvements were achieved without resorting to structurally complex or computationally intensive control architectures, thereby preserving the simplicity and practicality of PID-based designs. The adoption of SFOA for parameter optimization enabled efficient global tuning of the nonlinear controller structure. Statistical analyses confirmed that SFOA consistently achieved the lowest cost function values with minimal variance, demonstrating strong convergence stability and robustness in a high-dimensional and multimodal optimization landscape. Extensive time-domain simulations further revealed that the optimized G-PID controller outperformed classical PID, FOPID, PID-F, PIDA, and higher-order differential controllers. In particular, a rise time of 0.0551 s, zero overshoot, and a settling time of 0.0830 s were achieved, surpassing all benchmark optimizers (including PSO, GWO, L-SHADE, and KLA) as well as recent state-of-the-art AVR designs reported between 2024 and 2025. These results confirm the controller’s ability to deliver faster dynamics, smoother voltage regulation, and enhanced stability relative to advanced contemporary approaches.

The applicability of the proposed SFOA-optimized G-PID controller to real-time AVR scenarios can be inferred from the presented simulation results. The consistently fast and overshoot-free transient responses indicate strong damping and stable closed-loop behavior, which are essential requirements for real-time voltage regulation. The bounded and smooth nature of the Gudermannian nonlinear mapping ensures well-regulated control action during large voltage deviations, thereby reducing the likelihood of actuator saturation or excessive control effort in practical implementations. Furthermore, the controller retains a standard PID-type structure and does not depend on fractional operators, fuzzy rule bases, or cascaded architectures, which simplifies digital realization and limits computational burden. Since the optimization process is carried out offline and the resulting controller operates with fixed parameters during runtime, the proposed approach is well suited for implementation on conventional digital control hardware.

Despite these advantages, certain limitations should be acknowledged. The results reported in this study are based on detailed MATLAB/Simulink simulations, and real-world implementation may be influenced by factors such as measurement noise, actuator saturation, computational delays, and quantization effects, which are not explicitly modeled. In addition, the inclusion of the Gudermannian nonlinear mapping introduces extra tuning parameters beyond those of a classical PID controller. Although these parameters are efficiently optimized using SFOA, the increased dimensionality may result in higher offline computational cost compared with traditional tuning approaches. Moreover, the AVR model employed relies on a standard linearized representation and does not explicitly consider magnetic saturation or severe nonlinear operating conditions. The present investigation is also limited to a single-machine AVR system, and extension to multi-machine or wide-area voltage regulation scenarios has not yet been addressed.

Accordingly, future research may focus on real-time implementation and experimental validation of the proposed controller on digital signal processors or embedded control platforms. Further performance enhancement may also be achieved by integrating the Gudermannian-based control framework with adaptive, predictive, or model-based control strategies to better handle deeper nonlinearities and stronger parameter variations. In addition, extending the proposed methodology to multi-area power systems, wide-area voltage regulation schemes, and renewable-dominated grids would provide valuable insight into its scalability and broader applicability. These directions offer promising opportunities for advancing nonlinear, metaheuristic-optimized voltage regulation strategies in next-generation power systems.

## Figures and Tables

**Table 1:** Summary of efforts in previous research.

Ref.	Controller Architecture	Optimization Algorithm	Advantage	Disadvantage	Achievement
[13]	Cascaded RPIDD^2^-FOPI	QWGBO	Ultra-fast transient response, state-of-the-art settling time, zero overshoot	Highly complex cascaded architecture; multi-component design	Achieves near-optimal rise time and the fastest settling time, guaranteeing zero overshoot
[35]	PI^λ1^I^λ2^D^μ1^D^μ2^	Mayfly Algorithm (MA)	Achieves zero overshoot and excellent speed: enhanced flexibility via 4 fractional orders	Highly complex 9-parameter fractional-order structure; high-dimensional tuning space	Achieves near-optimal rise time and ultra-fast settling time, with zero overshoot
[36]	FOPIDD^2^	m-ARO	Attains zero overshoot and ultra-fast settling time with increased flexibility	Complex 6-parameter FOPIDD^2^ structure; high dimensionality	Very fast rise time and ultra-fast settling time, maintaining zero overshoot
[37]	PIDD^2^ (Master/Slave)	L-RUN	Zero overshoot and ultra-fast response using integer-order components only	Relies on complex master/slave tuning approach for robust parameter determination.	Very fast rise time and ultra-fast settling time, delivering zero overshoot
[12]	Fuzzy FOPI+FOPD+I	TLBO	Integrates fuzzy logic for high robustness and real-time adaptability to uncertainties	Extremely high complexity (9-parameter hybrid structure) and needs fuzzy rule base design	Achieves moderate rise and settling times, significantly reducing but not eliminating overshoot
[30]	TI^λ^DND^2^N^2^	Equilibrium Optimizer (EO)	Extremely fast rise and settling times, highly robust against disturbances and parameter change	Exhibits low but non-zero overshoot; highly augmented fractional/tilt structure.	Achieves ultra-fast settling time and very fast rise time, with minimal overshoot.
[31]	FOPID	MP-SEDA	Enhanced flexibility over basic PID, achieving fast response for a pure FOPID structure.	Exhibits moderate overshoot; higher tuning complexity due to fractional orders	Fast response time and reduced settling time, but with a perceptible overshoot
[32]	Multi-term FOPID	Rao Algorithm	Very low overshoot (minimal peaking), good balance of speed and stability	Settling time is noticeably slower compared to the leading ultra-fast cascaded methods	Fast rise and settling times, characterized by extremely minimal overshoot
[33]	PID-F	Symbiotic Organism Search (SOS)	Achieves near-zero overshoot (minimal peaking); structurally simpler than fractional methods.	Exhibits slow settling time; response is significantly slower than ultra-fast designs	Slowest response among the compared zero/near-zero overshoot methods, but with near-zero overshoot
[34]	LOA-FOPID	IABC	Uses fractional order for better robustness and fine control flexibility	Exhibits the largest overshoot among the advanced FOPIDs; slower response time	Slower rise and settling times, accompanied by the highest reported overshoot among advanced methods
Proposed Study	Gudermannian-PID (G-PID)	SFOA	Introduces analytically derived, smooth non-linearity for enhanced adaptability. Uses a superior metaheuristic for global tuning.	Requires tuning of an internal non-linear scaling parameter in addition to PID gains.	Target: Achieve performance comparable to the fastest, zero-overshoot cascaded designs, but with a simpler functional structure.

**Table 2:** Pseudo code for SFOA optimization technique.

1	$\mathrm{Initialize}\;\mathrm{population}\;X_{i}\;\left(i=1,\ldots ,N\right)$ uniformly within the bounds ([lb,ub]).
2	$\mathrm{Evaluate}\;\mathrm{the}\;\mathrm{fitness}\;\mathrm{of}\;\mathrm{each}\;\mathrm{starfish}:\;F_{i}=f\left(X_{i}\right)$.
3	$\mathrm{Identify}\;\mathrm{the}\;\mathrm{best}\;\mathrm{solution}\;X_{best}$
4	For iteration (t=1$)\;\mathrm{to}\;T_{max}$:
5	Generate a random number r∈(0,1).
6	If (*r* < 0.5): (*Exploration Phase*)
7	If (*D* > 5): apply the five-dimensional search pattern.
8	Randomly select five dimensions (*p*).
9	$\mathrm{Update}:\;Y_{i,p}^{\left(t\right)}=X_{i,p}^{\left(t\right)}\pm \alpha_{1}\left(X_{best,p}^{\left(t\right)}-X_{i,p}^{\left(t\right)}\right)\Phi \left(\theta \right)$
10	$\mathrm{Apply}\;\mathrm{boundary}\;\mathrm{control}\;\mathrm{to}\;\mathrm{each}\;Y_{i}$.
11	Else (*D* ≤ 5): apply the unidimensional search pattern.
12	Select one dimension (*p*) and two random starfish (*k_1_*) and (*k_2_*).
13	$\mathrm{Update}:\;Y_{i,p}^{\left(t\right)}=E_{t}X_{i,p}^{\left(t\right)}+A_{1}\left(X_{k_{1},p}^{\left(t\right)}-X_{i,p}^{\left(t\right)}\right)+A_{2}\left(X_{k_{2},p}^{\left(t\right)}-X_{i,p}^{\left(t\right)}\right)$
14	$\mathrm{Apply}\;\mathrm{boundary}\;\mathrm{control}\;\mathrm{to}\;\mathrm{each}\;Y_{i}$.
15	Else (*r* ≥ 0.5): (*Exploitation Phase*)
16	$\mathrm{Compute}\;\mathrm{distances}:\;d_{m}=X_{best}-X_{m}$ , for m=1,…,5
17	$\mathrm{Update}\;\mathrm{each}\;\mathrm{starfish}:\;Y_{i}^{\left(t\right)}=X_{i}^{\left(t\right)}+r_{1}d_{m_{1}}+r_{2}d_{m_{2}}$
18	$\mathrm{Apply}\;\mathrm{boundary}\;\mathrm{control}\;\mathrm{to}\;\mathrm{each}\;Y_{i}$
19	$\mathrm{Apply}\;\mathrm{regeneration}\;\mathrm{to}\;\mathrm{the}\;\mathrm{worst}\;\mathrm{starfish}:\;Y_{i}^{\left(t\right)}=e^{-\frac{t\times N}{T_{max}}}X_{i}^{\left(t\right)}$
20	$\mathrm{Evaluate}\;\mathrm{updated}\;\mathrm{fitness}\;\mathrm{values}\;F_{i}=f\left(Y_{i}\right)$
21	$\mathrm{Update}\;\mathrm{population}:\;X_{i}⟵Y_{i}$
22	$\mathrm{Update}\;X_{best}$ if a better solution is found.
23	End For
24	$\mathrm{Return}\;\mathrm{the}\;\mathrm{best}\;\mathrm{solution}\;X_{best}$ and its fitness.

**Table 3:** Main elements of AVR system.

Infrastructure	Approved Gain Ranges	Approved Time Ranges	Gain	Time
Amplifier	$10\;{\leq K}_{a}\leq 40$	$0.02\;{\leq T}_{a}\leq 0.1$	$K_{a}=10$	$T_{a}=0.1$
Exciter	$1\;{\leq K}_{e}\leq 10$	$0.4\;{\leq T}_{e}\leq 1$	$K_{E}=1$	$T_{e}=0.4$
Generator	$0.7\;{\leq K}_{g}\leq 1$	$1\;{\leq T}_{G}\leq 2$	$K_{g}=1$	$T_{g}=1$
Sensor	$0.7\;{\leq K}_{s}\leq 1$	$0.001\;{\leq T}_{s}\leq 0.06$	$K_{s}=1$	$T_{s}=0.01$

**Table 4:** Parameter bounds for the optimization problem.

Bound	$K_{P}$	$K_{I}$	$K_{D}$	η	$\tau_{1}$	$G_{1}$	$\tau_{2}$	$G_{2}$
Lower	0.1	0.01	0.1	5	0.05	0.02	0.02	0.02
Upper	20	1	10	1000	10	5	5	5

**Table 5:** Control parameters of SFOA, KLA, L-SHADE, GWO and PSO.

Algorithm	Reference	Parameter	Value
SFOA	[42]	$\mathrm{Algorithmic}\;\mathrm{parameter}\;(G_{P}$)	0.5
KLA	[43]	Without control parameters	−
L-SHADE	[44]	$\mathrm{Crossover}\;\mathrm{rate}\;(M_{CR}$$),\;\mathrm{scaling}\;\mathrm{factor}\;(M_{F}$)	0.5, 0.5
GWO	[7]	Convergence constant (a)	Decreases linearly from 2 to 0
PSO	[45]	$\mathrm{Inertia}\;\mathrm{weights}\;(w_{min}$$\;\mathrm{and}\;w_{max}$$),\;\mathrm{accelerating}\;\mathrm{coefficients}\;(c_{1}$$\;\mathrm{and}\;c_{2}$)	0.6, 0.9, 2, 2

**Table 6:** Statistical performance metrics of the evaluated algorithms.

Algorithm	Best	Worst	Average	SD
SFOA	1.0241E−02	1.1613E−02	1.0888E−02	4.0055E−04
KLA	1.0773E−02	1.2278E−02	1.1456E−02	3.5010E−04
L-SHADE	1.0655E−02	1.1977E−02	1.1189E−02	3.6076E−04
GWO	1.1741E−02	1.3975E−02	1.2690E−02	6.0568E−04
PSO	1.1158E−02	1.2880E−02	1.1933E−02	5.0425E−04

**Table 7:** Comparison of optimized controller parameters across optimization methods.

Algorithm	$K_{P}$	$K_{I}$	$K_{D}$	η	$\tau_{1}$	$G_{1}$	$\tau_{2}$	$G_{2}$
SFOA	6.7184	0.5812	4.0806	983.8587	4.5068	0.9032	0.0556	1.6840
KLA	6.3726	0.7403	3.9675	894.5021	4.6930	0.9664	0.0423	2.3945
L-SHADE	9.3261	0.8760	4.4792	814.7676	3.8887	0.8522	0.0359	2.1488
GWO	4.3084	0.9142	4.6478	998.3368	8.8470	1.6975	0.0641	3.8489
PSO	3.7166	0.5562	1.8472	861.8583	5.1630	1.6949	0.0910	2.3792

**Table 8:** Transient performance metrics of controllers optimized by SFOA and benchmark methods.

Algorithm	Rise Time (s)	Overshoot (%)	Settling Time (s)
SFOA	0.0551	0	0.0830
KLA	0.0581	0	0.0874
L-SHADE	0.0511	0.1224	0.0780
GWO	0.0667	0.2163	0.0949
PSO	0.0615	0.0456	0.0911

**Table 9:** Controller formulations and optimal parameter values reported in the literature.

Controller Design Method	Controller Transfer Function	Parameter Values
TSA-optimized PID	$K_{P}+\frac{K_{I}}{s}+K_{D}s$	$K_{P}=1.1281$ $,\;K_{I}=0.9567$ $,\;K_{D}=0.5671$
AOA-optimized PID-F	$K_{P}+\frac{K_{I}}{s}+K_{D}\frac{Ns}{s+N}$	$K_{P}=0.6729$ $,\;K_{I}=0.6212$ $,\;K_{D}=0.2668$ , N=701.7538
HGSO-optimized FOPID	$K_{P}+\frac{K_{I}}{s^{\lambda }}+K_{D}s^{\mu }$	$K_{P}=2.6632$ $,\;K_{I}=1.1314$ $,\;K_{D}=0.4559$ , λ=1.2689 , μ=1.3663
WOA-optimized PIDA	$\frac{K_{A}s^{3}+K_{D}s^{2}+K_{P}s+K_{I}}{s^{3}+\alpha s^{2}+\beta s}$	$K_{P}=777.401$ $,\;K_{I}=397.741$ $,\;K_{D}=500.652$ $,\;K_{A}=103.02$ , α=550.118 , β=915.041
FWWOA-optimized PIDD^2^	$K_{P}+\frac{K_{I}}{s}+K_{D}s+K_{DD}s^{2}$	$K_{P}=2.9743$ $,\;K_{I}=1.9827$ $,\;K_{D}=1.0705$ $,\;K_{DD}=0.0790$

**Table 10:** Comparison of rise time, overshoot, and settling time for the proposed controller and benchmark designs.

Controller Design Method	Rise Time (s)	Overshoot (%)	Settling Time (s)
SFOA-optimized G-PID (proposed)	0.0551	0	0.0830
TSA-optimized PID	0.1310	15.570	0.7580
AOA-optimized PID-F	0.2549	1.9977	0.3816
HGSO-optimized FOPID	0.0892	2.8626	0.4257
WOA-optimized PIDA	0.3280	2.0000	0.4530
FWWOA-optimized PIDD^2^	0.0848	0.0033	0.1484

**Table 11:** Comparative list of recent optimization-based controller designs reported in the literature.

Method No.	Reference	Year	Controller Type	Optimizer
1	[51]	2025	Fuzzy PIDF+FOPD	Sand cat swarm optimization
2	[52]	2025	Extended PIDA	Snake algorithm
3	[53]	2025	PIDF+Fuzzy PIDF	Teaching-learning-based optimization
4	[54]	2025	PID	Improved coati optimization algorithm
5	[55]	2025	FOPID	Artificial hummingbird algorithm
6	[56]	2024	Fast and robust SMC	Grey wolf optimization
7	[57]	2024	V-Tiger-PID	Pessen integral rule
8	[58]	2024	Sigmoid-based FOPID	Dandelion optimizer
9	[59]	2024	FHODFC	Honey badger algorithm
10	[60]	2024	PIDD^α^	Manta ray foraging optimization

## Data Availability

All related data are presented within the manuscript.
